# Supplementary material for: ZBTB18 regulates cytokine expression and affects microglia/macrophage recruitment and commitment in glioblastoma
Source: Commun Biol. 2024 Nov 8;7:1472. doi: 10.1038/s42003-024-07144-y (PMC11549471; doi:10.1038/s42003-024-07144-y)
Supplement: Supplementary file 2 — Supplementary Information [file 42003_2024_7144_MOESM2_ESM.pdf]

**Supplementary Information**

# ZBTB18 regulates cytokine expression and affects microglia/macrophage recruitment and commitment in glioblastoma

Roberto Ferrarese[§], Kevin Joseph[§], Geoffroy Andrieux[§], Ira Verena Haase, Francesca Zanon, Eva Kling, Annalisa Izzo, Eyleen Corrales, Marius Schwabenland, Marco Prinz, Vidhya Madapusi Ravi, Melanie Boerries, Dieter Henrik Heiland, and Maria Stella Carro[*]

*Corresponding author: Maria Stella Carro, Department of Neurosurgery, University Medical Center Freiburg, Breisacher Straße 64, 79106 Freiburg, Germany (maria.carro@uniklinik-freiburg.de)

§ These authors equally contributed to this work

Supplementary Figure 1

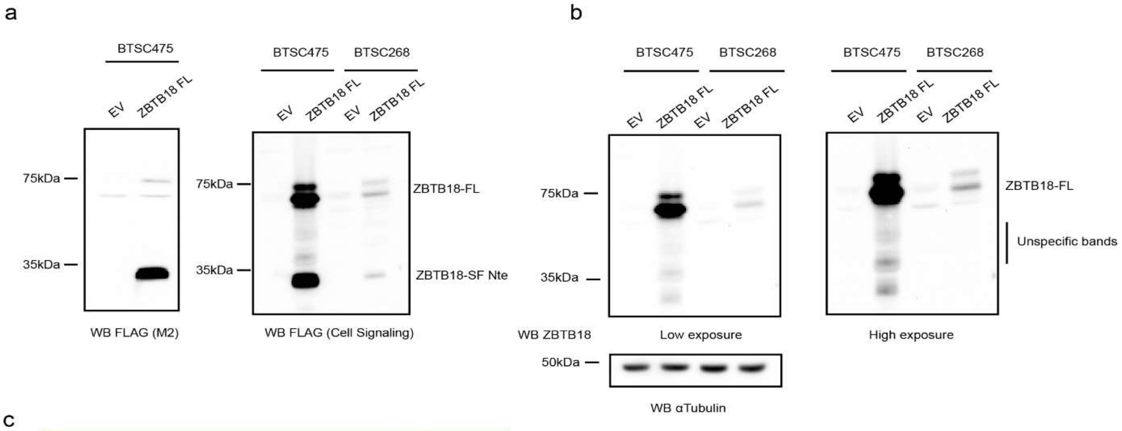


c

| Group | Genes |
|---|---|
| BTSC233;JX6;SNB19;BTSC475 | GDF15 |
| BTSC233;JX6;SNB19;BTSC475 | CXXC5 |
| BTSC233;JX6;SNB19;BTSC475 | PDGFRA |
| BTSC233;JX6;SNB19;BTSC475 | S100A6 |
| BTSC233;JX6;SNB19;BTSC475 | CMTM7 |
| JX6;SNB19;BTSC268;BTSC475 | CXCL14 |
| JX6;SNB19;BTSC268;BTSC475 | CCL2 |
| BTSC233;JX6;SNB19;BTSC268;BTSC475 | CX3CL1 |

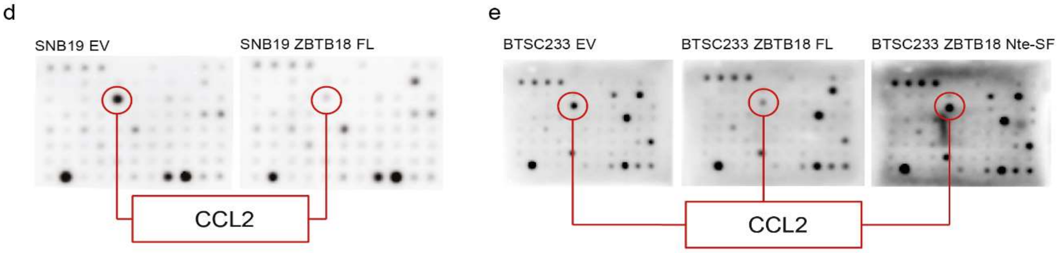


f

| Sample | | CCL2 |
|---|---|---|
| SNB19 | EV | 1 |
| | ZBTB18 FL | 0,11 |
| BTSC233 | EV | 1 |
| | ZBTB18 FL | 0,18 |
| | ZBTB18 Nte-SF | 1,08 |

**Supplementary Figure 1. ZBTB18 halts the expression and secretion of cytokines in GBM cells.** (a-b) Western blot analysis of ZBTB18 ectopic expression in primary GBM patient-derived BTSC268 and in BTSC475 cells using a FLAG (a) or an anti-ZBTB18 antibody (b). αTubulin is included as a loading control. (c) List of downregulated cytokine-coding genes identified in at least four out of five ZBTB18 overexpression studies, by Venn analysis. (d-e) Cytokine antibody array of SNB19 cells expressing control EV or ZBTB18 FL (d), and of BTSC233 cells expressing control EV, ZBTB18 FL, or ZBTB18 Nte-SF (e); the spot corresponding to MCP1/CCL2 is highlighted in red. (f) Quantification of the signal detected in (d) and (e).

Supplementary Figure 2

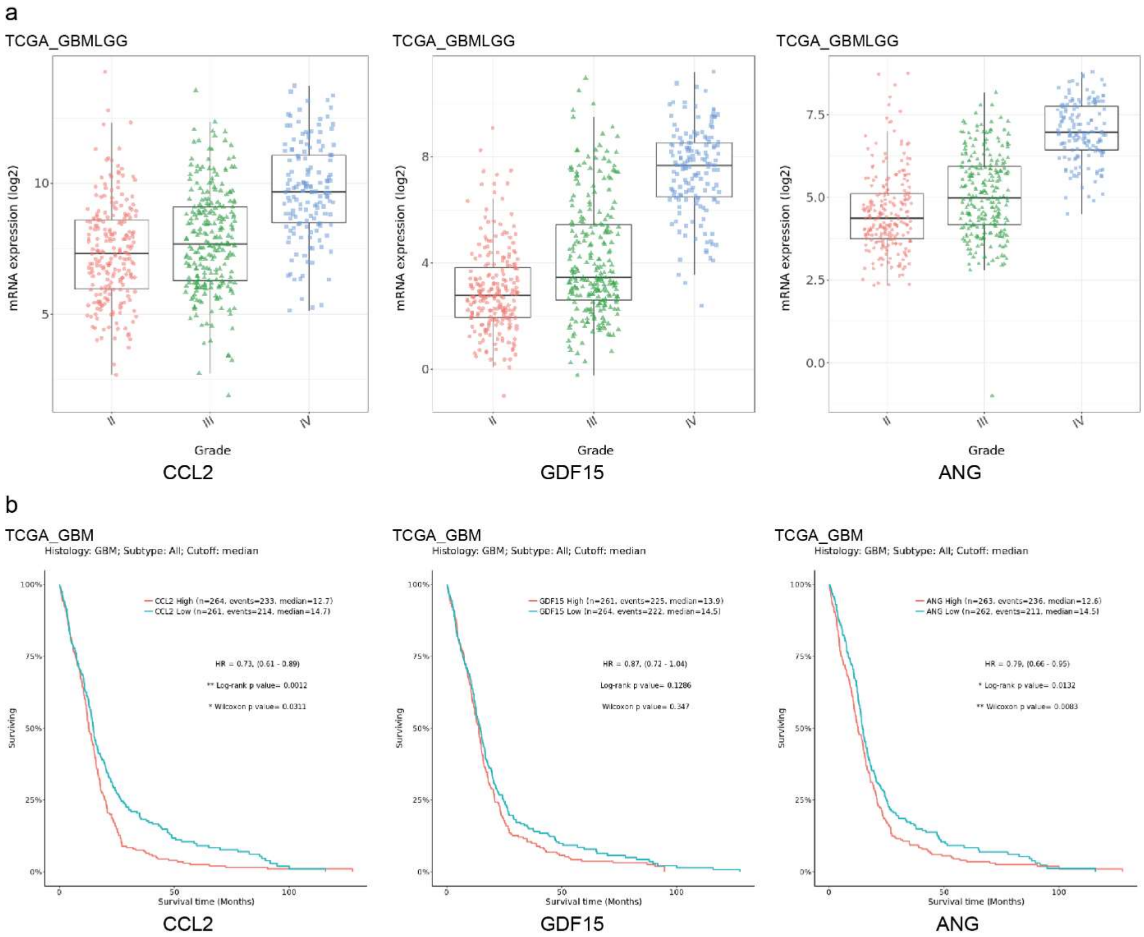


**Supplementary Figure 2. Gliovis analysis of CCL2, GDF15, or ANG association with tumor grade and survival in the TCGA_GBMLGG dataset.** (a) GlioVis analysis of *CCL2*, *GDF15*, and *ANG* expression in different-grade gliomas from the TCGA_GBMLGG dataset. (b) Kaplan-Meier estimator survival analysis of patients with high or low expression of *CCL2*, *GDF15*, or *ANG* in the TCGA_GBM dataset.

Supplementary Figure 3

a

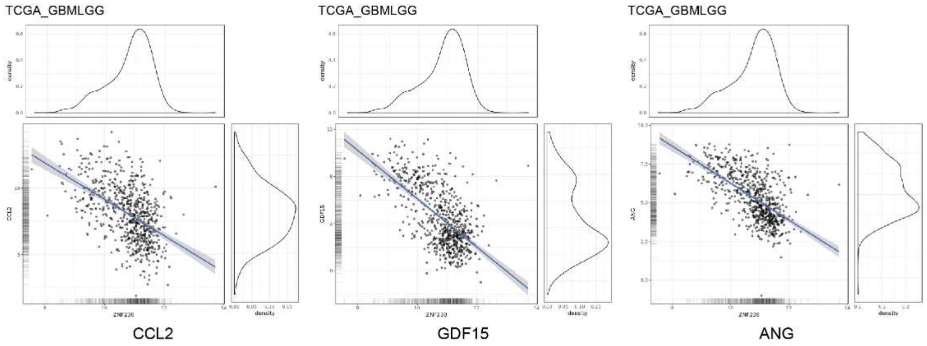


b

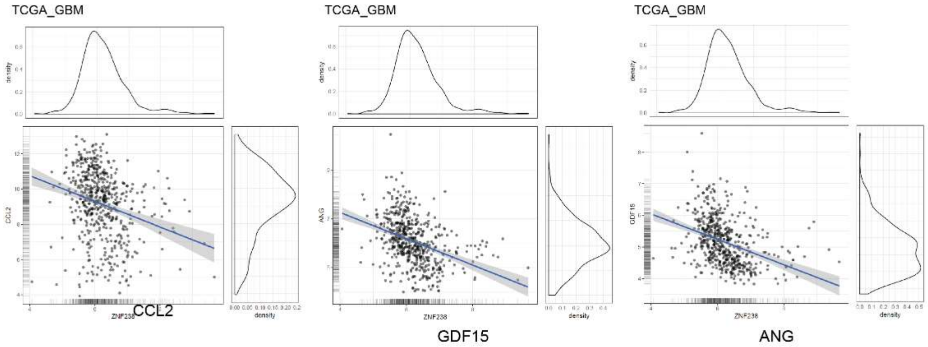


c

| | TCGA_GBM | | TCGA_GBMLGG | |
|---|---|---|---|---|
| ZBTB18 correlation vs. | r | p | r | p |
| CCL2 | -0,26 | e-6,26 | -0,5 | 0 |
| GDF15 | -0,37 | e-9,27 | -0,62 | 0 |
| ANG | -0,39 | e-9,68 | -0,61 | 0 |

**Supplementary Figure 3. *ZBTB18* inversely correlates with *CCL2*, *GDF15* or *ANG* expression in the TCGA datasets.** (a) Pearson correlation analysis between *ZBTB18* and *CCL2*, *GDF15*, or *ANG* expression in the TCGA_GBMLGG dataset. (b) Pearson correlation analysis between *ZBTB18* and *CCL2*, *GDF15*, or *ANG* expression in the TCGA_GBM dataset. (c) Summary table of the statistics shown in (a) and in (b).

Supplementary Figure 4

a

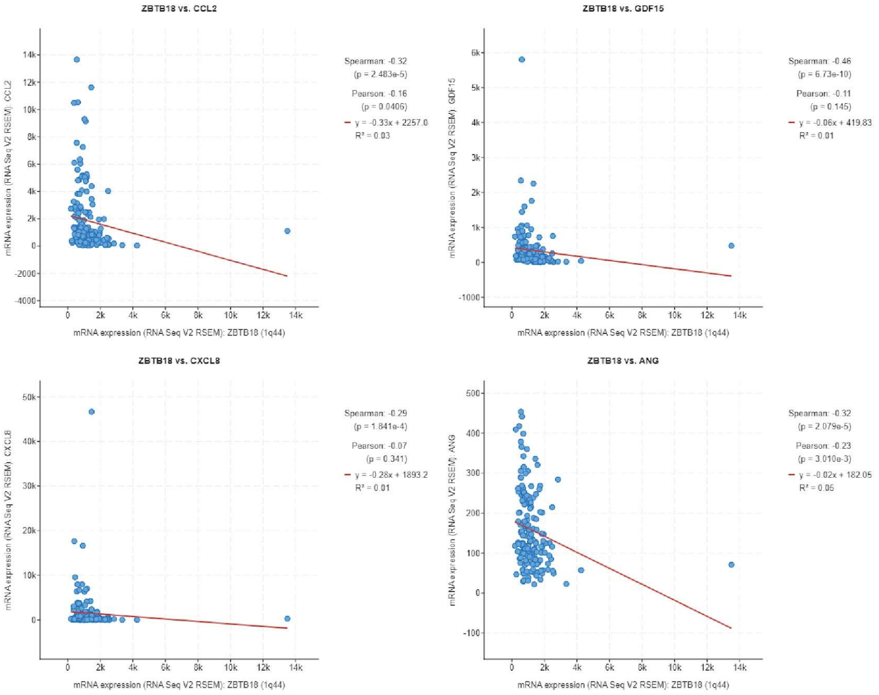


b

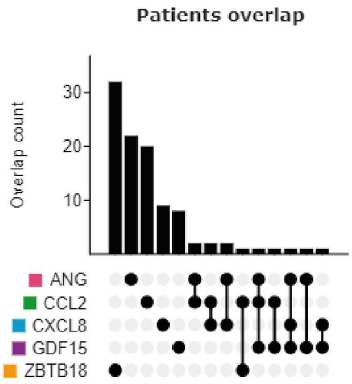


c

| A | B | Neither | A Not B | B Not A | Both | Log2 Odds Ratio | p-Value | q-Value | Tendency |
|---|---|---|---|---|---|---|---|---|---|
| GDF15 | ANG | 489 | 10 | 26 | 3 | 2.496 | 0.029 | 0.164 | Co-occurrence |
| CXCL8 | ANG | 487 | 12 | 26 | 3 | 2.227 | 0.043 | 0.164 | Co-occurrence |
| GDF15 | CXCL8 | 502 | 11 | 13 | 2 | 2.812 | 0.049 | 0.164 | Co-occurrence |
| CCL2 | GDF15 | 490 | 25 | 11 | 2 | 1.833 | 0.139 | 0.299 | Co-occurrence |
| CCL2 | CXCL8 | 488 | 25 | 13 | 2 | 1.586 | 0.176 | 0.299 | Co-occurrence |
| CCL2 | ANG | 475 | 24 | 26 | 3 | 1.191 | 0.179 | 0.299 | Co-occurrence |
| ZBTB18 | ANG | 466 | 33 | 29 | 0 | <-3 | 0.245 | 0.349 | Mutual exclusivity |
| ZBTB18 | CXCL8 | 480 | 33 | 15 | 0 | <-3 | 0.614 | 0.768 | Mutual exclusivity |
| ZBTB18 | CCL2 | 469 | 32 | 26 | 1 | -0.827 | 1.000 | 1.000 | Mutual exclusivity |
| ZBTB18 | GDF15 | 482 | 33 | 13 | 0 | <-3 | 1.000 | 1.000 | Mutual exclusivity |

**Supplementary Figure 4. Correlation analysis using cBioPortal** (a) cBioPortal co-expression analysis between *ZBTB18* and *CCL2*, *GDF15*, *CXCL8*, or *ANG* in samples from the Glioblastoma multiforme (TCGA, Firehose Legacy) dataset. (b) cBioPortal patients' overlap analysis for the listed genes within the Glioblastoma multiforme (TCGA, Firehose Legacy) dataset. (c) cBioPortal mutual exclusivity analysis between the genes indicated in the first two columns in samples from the Glioblastoma multiforme (TCGA, Firehose Legacy) dataset.

Supplementary Figure 5

a

| hPDI | start | end | width | seq | chr | gene | TSS | distance |
|---|---|---|---|---|---|---|---|---|
| 1 | 34255613 | 34255621 | 8 | CCAGCTGT | chr17 | CCL2 | 34255247 | -366 |
| 2 | 34258452 | 34258460 | 8 | GCAGATGT | chr17 | CCL2 | 34255247 | -3205 |
| 3 | 34260115 | 34260123 | 8 | ACAGATGT | chr17 | CCL2 | 34255247 | -4868 |
| 4 | 18382244 | 18382252 | 8 | ACAGATGT | chr19 | GDF15 | 18386148 | 3904 |
| 5 | 18386044 | 18386052 | 8 | CCAGCTGT | chr19 | GDF15 | 18386148 | 104 |
| 6 | 18389042 | 18389050 | 8 | ACAGCTGT | chr19 | GDF15 | 18386148 | -2894 |
| 7 | 18390264 | 18390272 | 8 | ACAGCTGT | chr19 | GDF15 | 18386148 | -4116 |
| 8 | 18390682 | 18390690 | 8 | ACAGCTGT | chr19 | GDF15 | 18386148 | -4534 |
| | | | | | | | | |
| HOCOMOCO | start | end | width | seq | chr | gene | TSS | distance |
| 1 | 34255612 | 34255623 | 11 | TCCAGCTGTG | chr17 | CCL2 | 34255247 | -365 |
| 2 | 34260114 | 34260125 | 11 | TACAGATGT[illegible] | chr17 | CCL2 | 34255247 | -4867 |
| 3 | 18382243 | 18382254 | 11 | TACAGATGTT | chr19 | GDF15 | 18386148 | 3905 |
| 4 | 18383881 | 18383892 | 11 | GCCAGGTGT[illegible] | chr19 | GDF15 | 18386148 | 2267 |
| 5 | 18386043 | 18386054 | 11 | CCCAGCTGTG | chr19 | GDF15 | 18386148 | 105 |

b

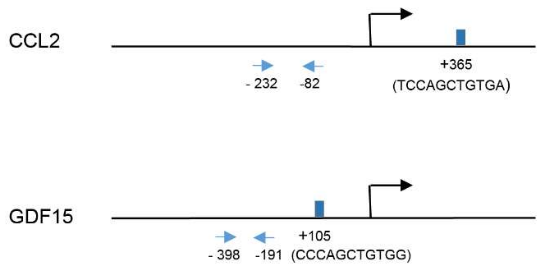


**Supplementary Figure 5. *CCL2* and *GDF15* promoters contain putative ZBTB18 binding motifs.** (a) Table showing ZBTB18 (ZNF238) motifs identified in *motifDB.* (b) Scheme of *CCL2* (upper panel) and *GDF15* (lower panel) promoters. The predicted binding site closest to the TSS is indicated. The position of the primers used in ChIP qPCR is also shown.

Supplementary Figure 6

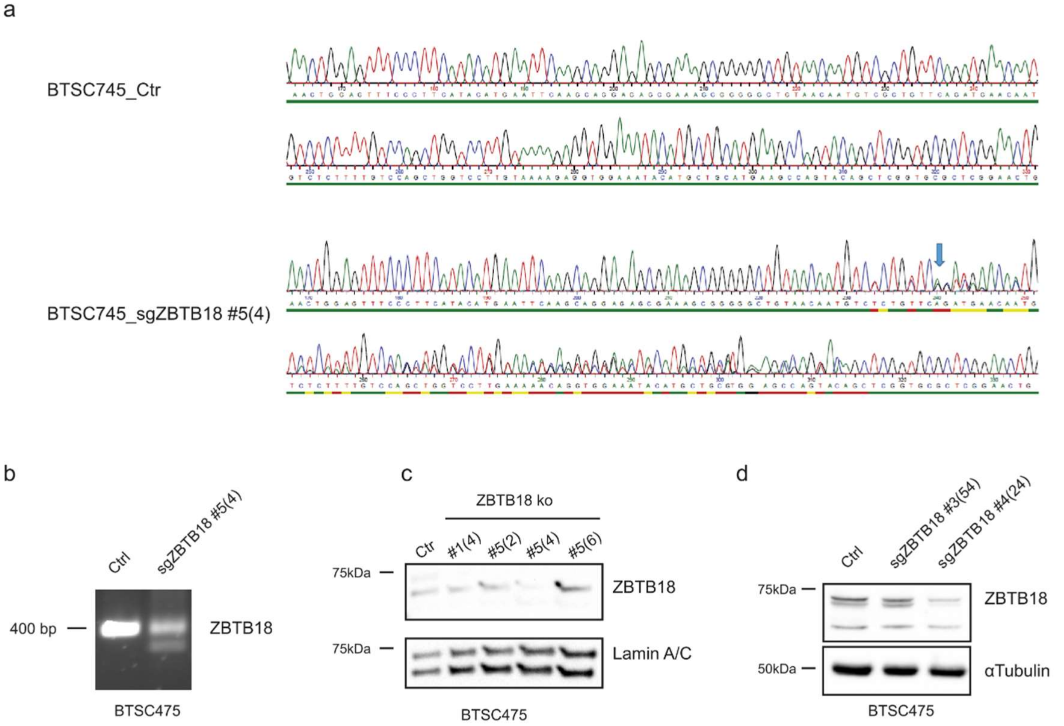


**Supplementary Figure 6. Analysis of ZBTB18 knockout in BTSC475.** (a) Electropherograms showing alteration of the *ZBTB18* locus upon CRISPR/Cas9 knockout with sgZBTB18#5 (clone 4). The *ZBTB18* locus of BTSC475-ctr is shown as reference. The mutated site is indicated by an arrow. (b) PCR analysis of *ZBTB18* locus in BTSC475-Ctr and BTSC475-sgZBTB18#5 (clones 4), surrounding the sgZBTB18#5. (c) Western blot analysis of ZBTB18 expression in nuclear extracts from BTSC475 Ctrl and in various clones of BTSC475 transduced with sgZBTB18#1 and #5. The sgZBTB18#5(4) is highlighted in bold. Lamin A/C is used as nuclear control. (d) Western blot analysis of ZBTB18 expression in total extracts from BTSC475 Ctrl and in two clones of BTSC475 transduced with sgZBTB18#3 and #4 as previously described [1]. The sgZBTB18#4(24) is highlighted in bold. αTubulin is used as a loading control.

Supplementary Figure 7

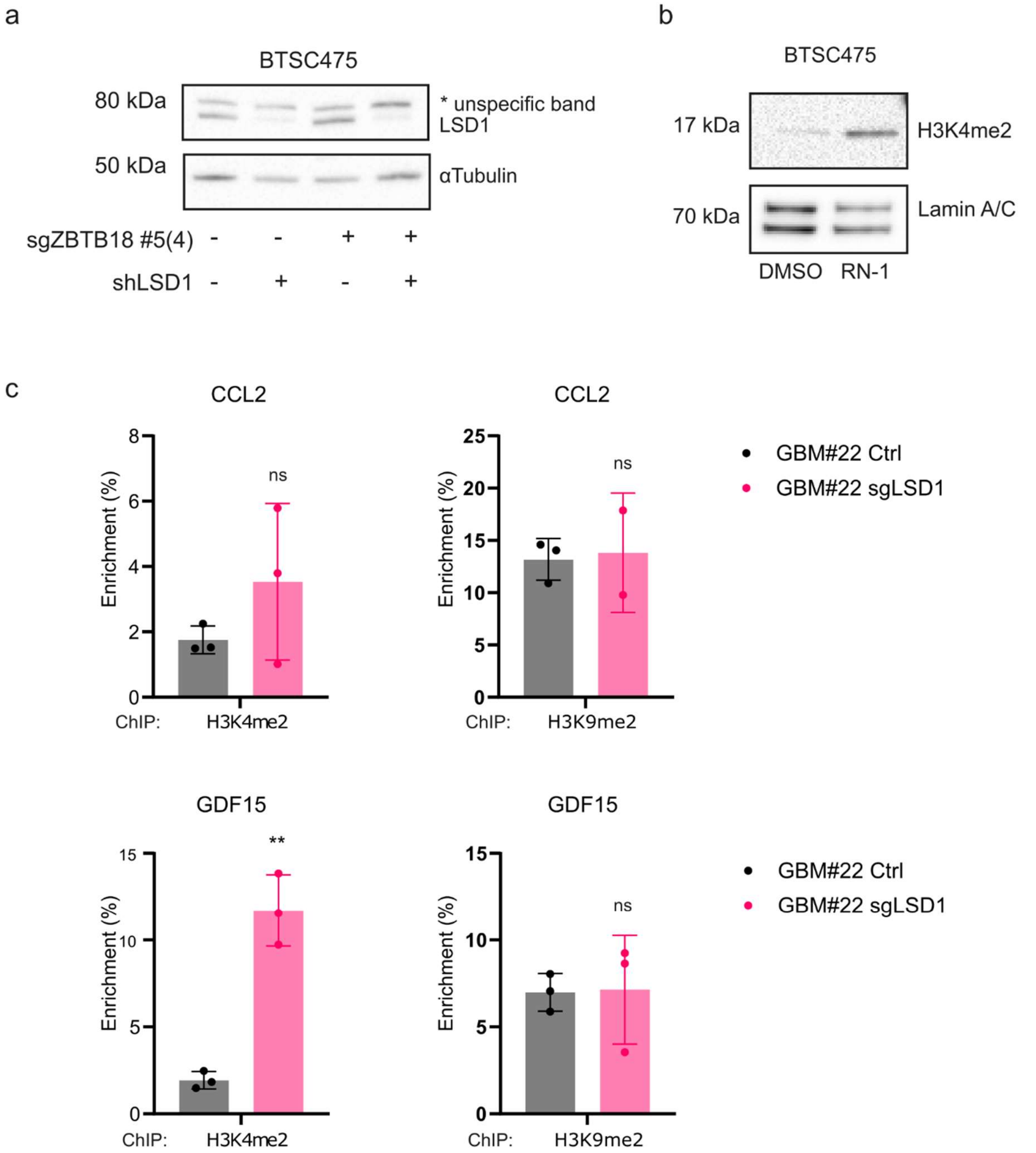


**Supplementary Figure 7. LSD1 knockout affects H3K4me2 at the GDF15 promoter.** (a) Western blot analysis of LSD1 expression in BTSC475 transduced as indicated. αTubulin is used as a loading control. (b) Western blot analysis of H3K4me2 mark upon treatment with the LSD1 inhibitor RN-1. (c) Enrichment of H3K4me2 and H3K9me2 ChIP at the *CCL2* (upper panels) and *GDF15* (lower panels) promoters, in GBM#22 sgLSD1. Graphs show the average q-RT PCR results of three independent ChIPs expressed in % input as indicated. Error bars ± s.d.; *$P < 0.05$ by a t test.

Supplementary Figure 8

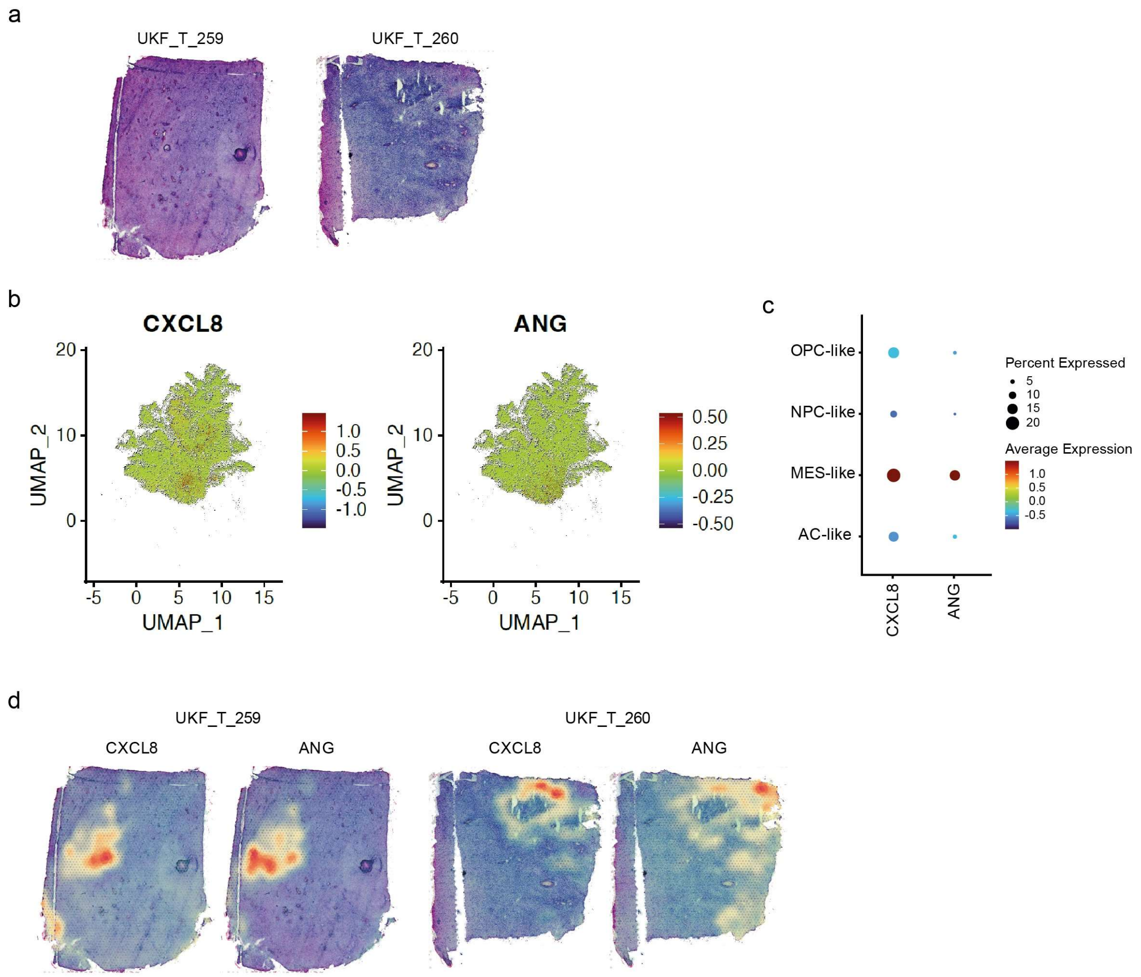


**Supplementary Figure 8. ANG and CXCL8 and ZBTB18 are complementary expressed within GBM subtypes.** (a) Reference micrographs for the stRNA spatial localization analysis shown in Figure 2D. (b) UMAP plot of *CXCL8* and *ANG* expression within the neoplastic population. The color bar represents the expression levels represented. (c) Dotplot visualization of the enrichment of *CXCL8* and *ANG* expression within neoplastic cell transcriptional programs. (d) stRNA visualization of the spatial localization of *CXCL8 and ANG* expression in two independent GBM sections.

Supplementary Figure 9

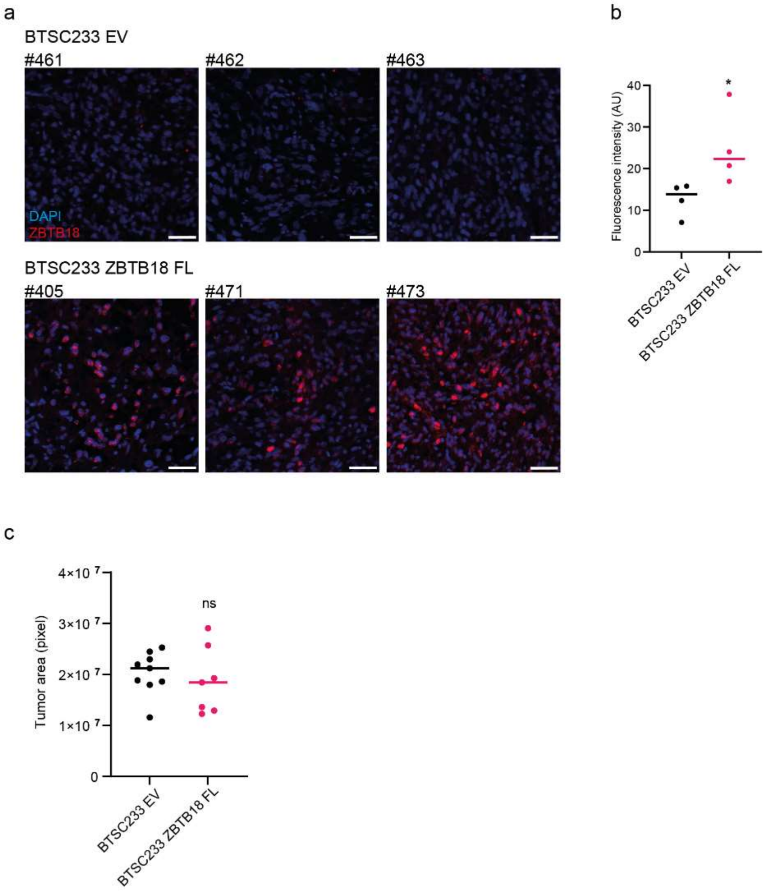


**Supplementary Figure 9. ZBTB18 expression and tumor size measurement in BTSC233 EV or BTSC233 ZBTB18 FL xenografts** (a) Representative micrographs of mouse brain sections with tumors derived from BTSC233 EV or BTSC233 ZBTB18 FL xenografts and stained with ZBTB18 rabbit polyclonal; scale bar: 50 µm. (b) Quantification of the immunostaining shown in (a); n=4 biological replicates; *P < 0.05 by a t test. (c) Quantification of tumor derived from BTSC233 EV or BTSC233 ZBTB18 FL xenografts based on H&E staining (representative staining were shown in [2]. n=9 (EV) and n=7 (ZBTB18-FL) biological replicates; ns (not significant).

Supplementary Figure 10

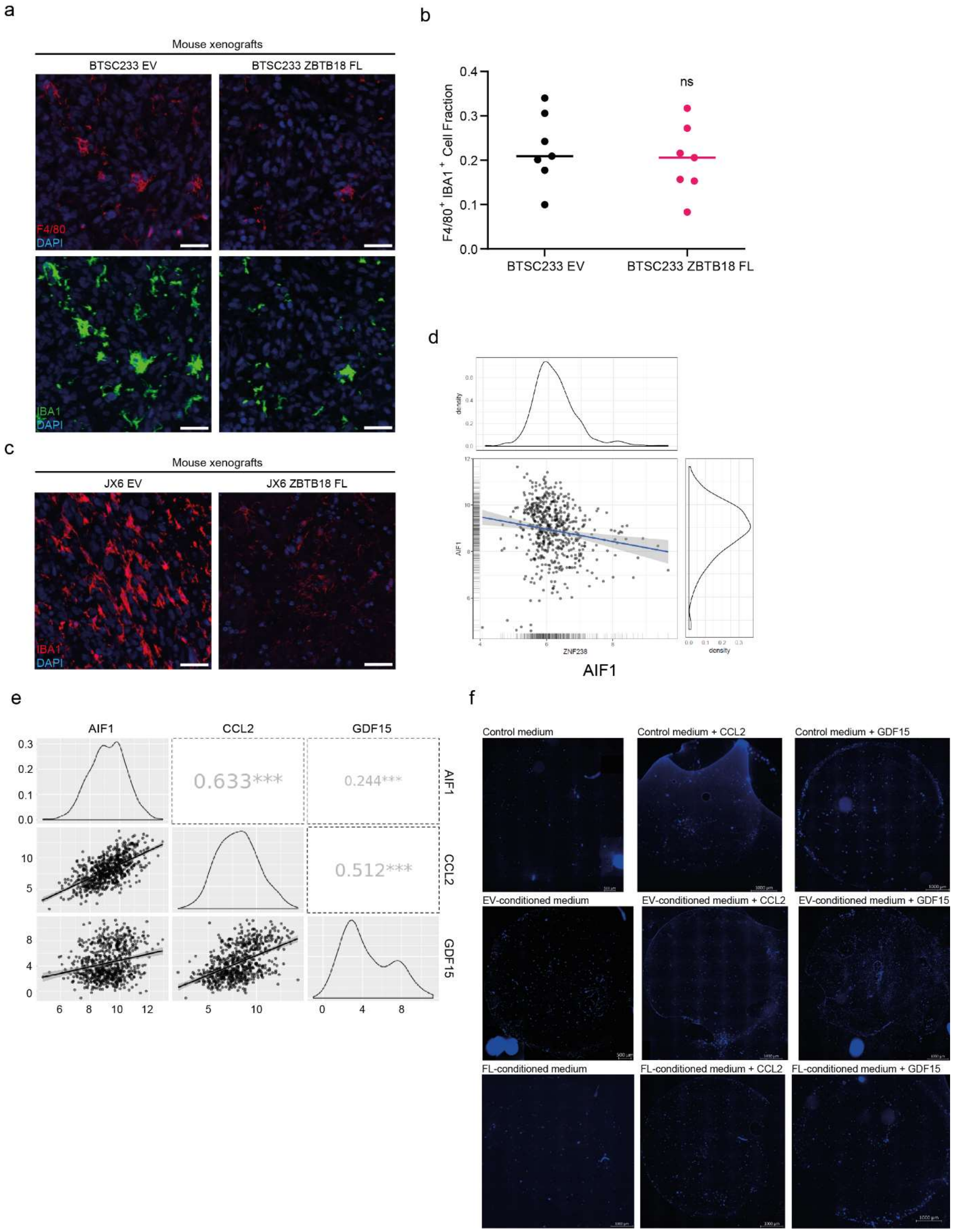


**Supplementary Figure 10. ZBTB18 expression in GBM cells impairs the GAM recruitment.** (a) Representative micrographs of mouse brain sections with tumors derived from BTSC233 EV or BTSC233 ZBTB18 FL xenografts and stained with F4/80 antibody (top panels) or IBA1 antibody (bottom panels); scale bar: 50 µm. (b) Quantification of the F4/80$^+$, IBA1$^+$ cell fraction in mouse brain sections represented in (a). (c) Representative micrographs of mouse brain sections with tumors derived from

JX6 EV or JX6 ZBTB18 FL xenografts and stained with IBA1 antibody; scale bar: 50 µm. (d) Pearson correlation analysis between *ZBTB18* and AIF1 or *ANG* expression in the TCGA_GBM dataset. r=-0,39; p=e-9,68. (e) Multiple Pearson correlation analysis between *AIF1*, *CCL2* and *GDF15*, expression in the TCGA_GBMLGG dataset. Lower diagonal: scatter plot with regression line; middle diagonal: density plot; upper diagonal: Pearson correlation coefficient with significance: *** < 0.001. (f) Representative micrographs of invading microglia corresponding to the analysis shown in Figure 4f.

Supplementary Figure 11

a

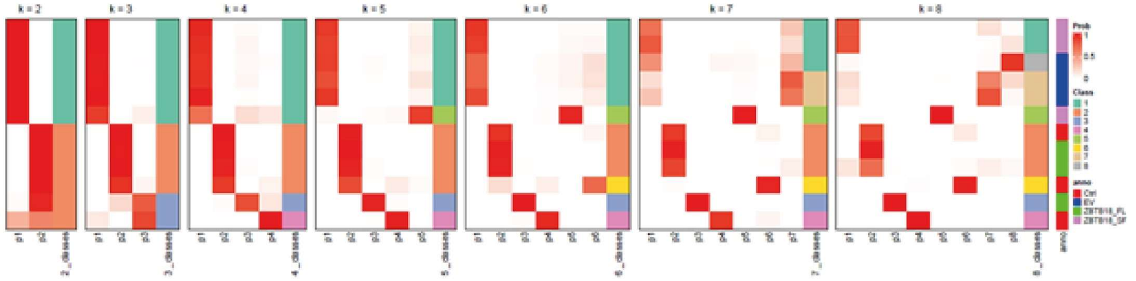

b

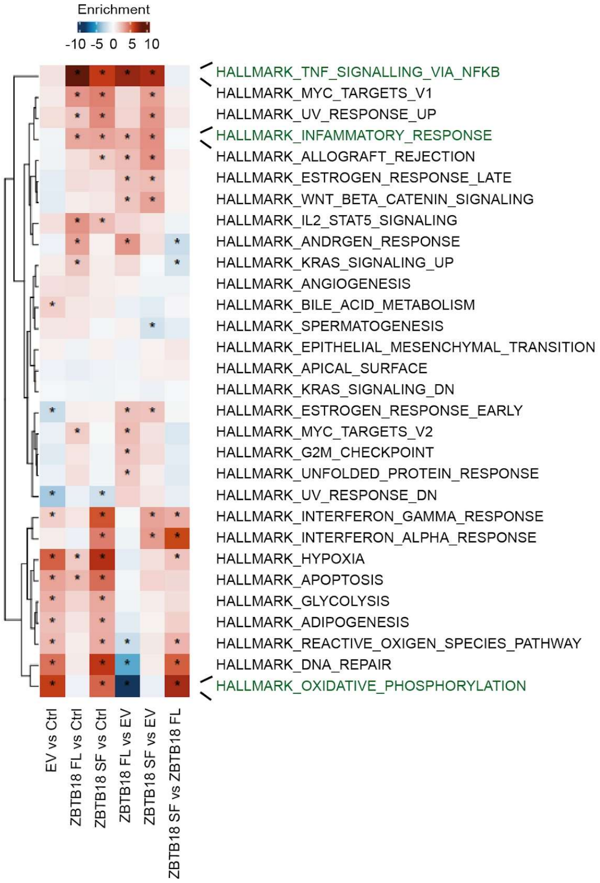


**Supplementary Figure 11. ZBTB18 expression in GBM cells induces a shift in microglia commitment.** (a) Consensus clustering portioning using skmeans (spherical k-means clustering) clustering on top 1, 5, 10, 25, 50 and 75% genes based on ATC with 100 repetitions. (b) Heatmap showing the up-or down-regulated hallmark signatures, analyzed by GSEA. Signatures related to the inflammatory response and oxidative phosphorylation are highlighted. Significant regulated signatures in the different group comparisons are highlighted with an asterisk (adj. p value < 0.05).

Supplementary Figure 12

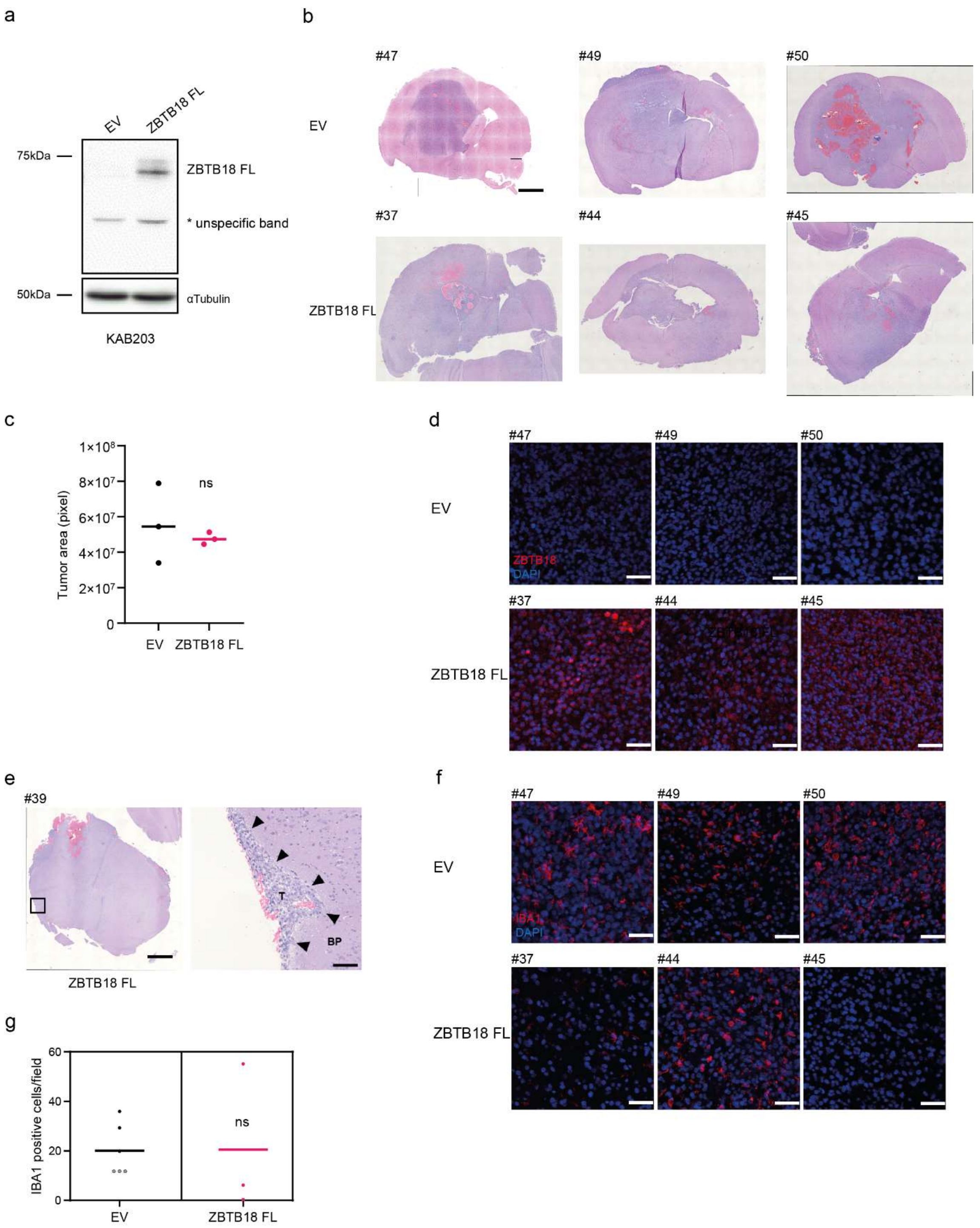


**Supplementary Figure 12. ZBTB18 affects MHCII expression in microglia *in vivo*.** (a) Western blot showing ZBTB18 FL expression in KAB203 cells transduced with control (EV) or ZBTB18 FL-expressing lentivirus. (b) Microphotographs of H&E-stained brain sections derived from C57BL/6 mice injected with KAB203 cells (transduced with

EV or ZBTB18 FL). Images were selected from tumors, which showed comparable characteristics in terms of size and location; scale bar: 1 mm. (c) Quantification of the area of the brain sections shown in (b); n=3. (d) Representative micrographs of mouse brain sections with tumors derived from KAB203 (transduced with EV or ZBTB18 FL) syngeneic mice and stained with ZBTB18 antibody; scale bar: 50 µm. (e) Representative H&E-stained brain section derived from KAB203-ZBTB18 injected mice, showing the presence of tumor cells along the meninges; scale bar: 1 mm. In the inset (scale bar 50 µm) the meninges are indicated by arrow heads. T= tumor, NB= brain parenchyma. (g) Representative micrographs of mouse brain sections with tumors derived from KAB203 (transduced with EV or ZBTB18 FL) syngeneic mice and stained with IBA1 antibody; scale bar: 50 µm. (h) Quantification of the immunostaining shown in (f); n=6 (EV), n=3 (ZBTB18 FL) biological replicates; *$P < 0.05$ by a t test. Dark dots correspond to the samples shown in (b) and (c).

Supplementary Figure 13

Uncropped blots

Figure 2g

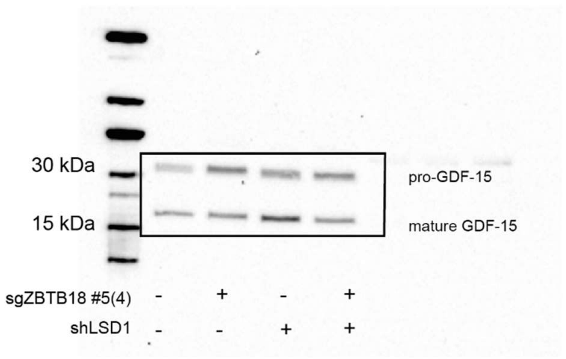


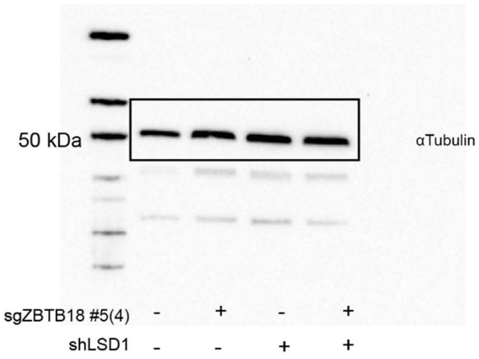


Supplementary Figure 1

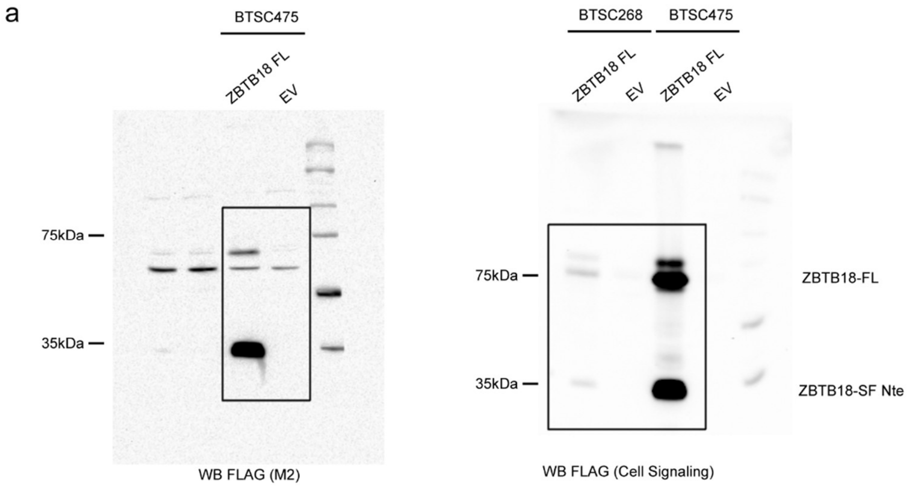


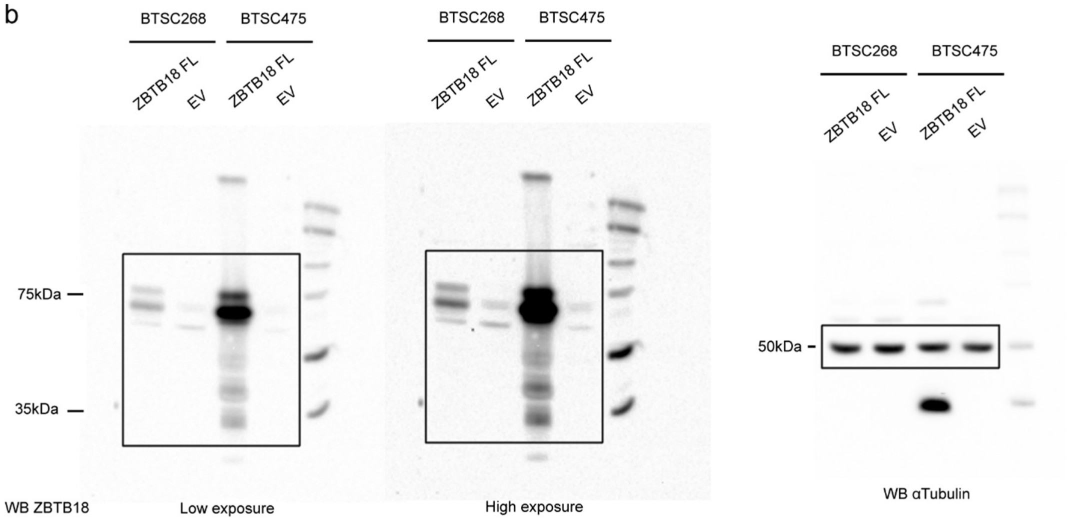

Supplementary Figure 14

Uncropped blots

Supplementary Figure 6b

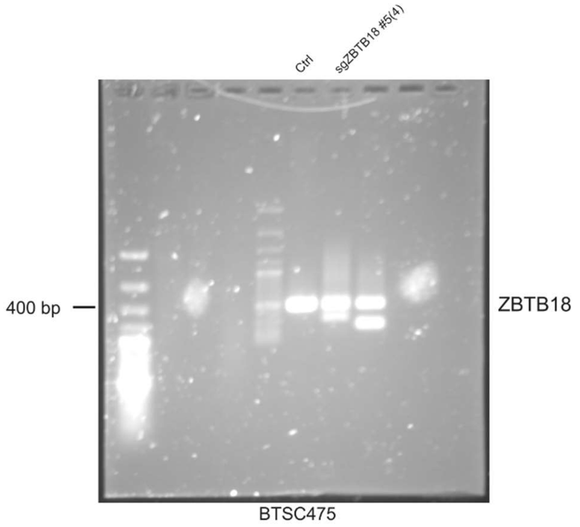


Supplementary Figure 6c

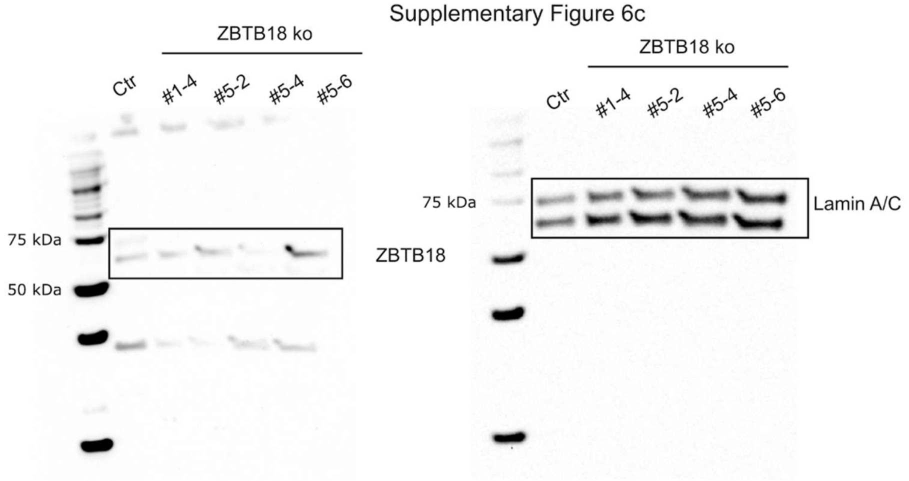


Supplementary Figure 6d

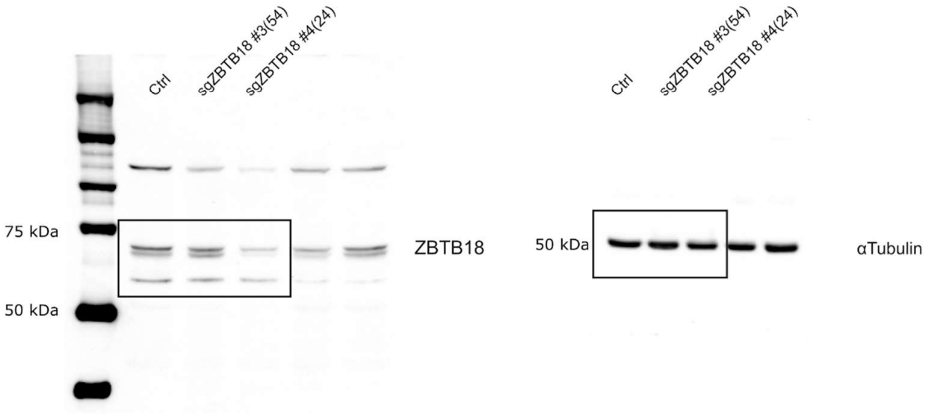

Supplementary Figure 14

Uncropped blots

## Supplementary Figure 7a

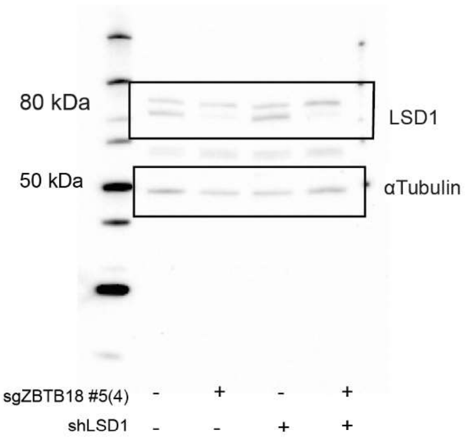


## Supplementary Figure 7b

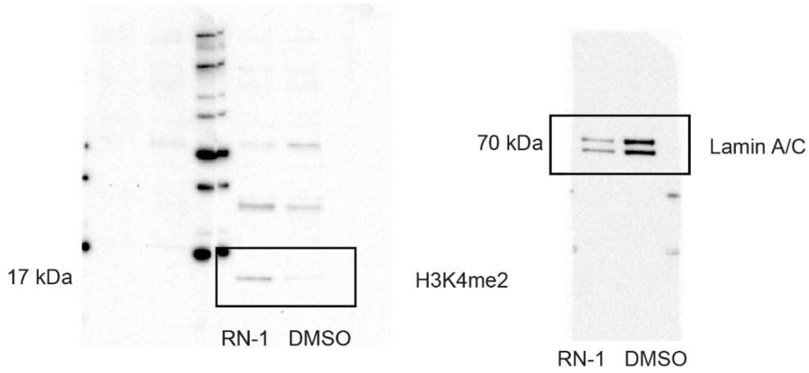


## Supplementary Figure 12a

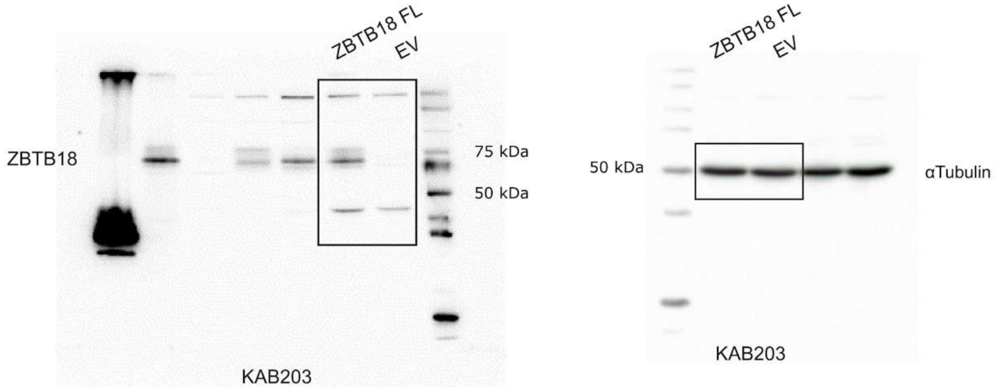

**Supplementary references**


1. Ferrarese R*, et al.* ZBTB18 inhibits SREBP-dependent lipid synthesis by halting CTBPs and LSD1 activity in glioblastoma. *Life Sci Alliance* **6**, (2023).

2. Fedele V*, et al.* Epigenetic Regulation of ZBTB18 Promotes Glioblastoma Progression. *Mol Cancer Res*, (2017).
