# Supplementary material for: ZBTB18 regulates cytokine expression and affects microglia/macrophage recruitment and commitment in glioblastoma
Source: Commun Biol. 2024 Nov 8;7:1472. doi: 10.1038/s42003-024-07144-y (PMC11549471; doi:10.1038/s42003-024-07144-y)
Supplement: Supplementary file 5 — reporting summary [file 42003_2024_7144_MOESM5_ESM.pdf]

nature portfolio

Corresponding author(s): Maria Stella Carro

Last updated by author(s): Oct 10, 2024

Reporting Summary

Nature Portfolio wishes to improve the reproducibility of the work that we publish. This form provides structure for consistency and transparency in reporting. For further information on Nature Portfolio policies, see our Editorial Policies and the Editorial Policy Checklist.

Statistics

For all statistical analyses, confirm that the following items are present in the figure legend, table legend, main text, or Methods section.

n/a	Confirmed	
☐	☒	The exact sample size (*n*) for each experimental group/condition, given as a discrete number and unit of measurement
☐	☒	A statement on whether measurements were taken from distinct samples or whether the same sample was measured repeatedly
☐	☒	The statistical test(s) used AND whether they are one- or two-sided<br>*Only common tests should be described solely by name; describe more complex techniques in the Methods section.*
☒	☐	A description of all covariates tested
☐	☒	A description of any assumptions or corrections, such as tests of normality and adjustment for multiple comparisons
☐	☒	A full description of the statistical parameters including central tendency (e.g. means) or other basic estimates (e.g. regression coefficient) AND variation (e.g. standard deviation) or associated estimates of uncertainty (e.g. confidence intervals)
☐	☒	For null hypothesis testing, the test statistic (e.g. *F*, *t*, *r*) with confidence intervals, effect sizes, degrees of freedom and *P* value noted<br>*Give P values as exact values whenever suitable.*
☒	☐	For Bayesian analysis, information on the choice of priors and Markov chain Monte Carlo settings
☒	☐	For hierarchical and complex designs, identification of the appropriate level for tests and full reporting of outcomes
☐	☒	Estimates of effect sizes (e.g. Cohen's *d*, Pearson's *r*), indicating how they were calculated

Our web collection on statistics for biologists contains articles on many of the points above.

Software and code

Policy information about availability of computer code

Data collection	Long read bulk sequencing data were collected using the MinKNOW software.
Data analysis	For RNAseq the following open sources software and code were used: FastQC (Andrews S. FastQC: a quality control tool for high throughput sequence data, from the web), Trimmomatic (v0.38) 1and STAR aligner.<br>For long read RNAseq the SpotON Flow Cell and MinKNOW software (Oxford Nanopore Technologies, Oxford, United Kingdom) were used. Basecalling was performed by Albacore implemented in the nanopore software.<br>Differential gene-expression analysis was performed using the limma (v3.54.0) R (v4.2.2) package. Altered pathways were identified using the Generally Applicable Gene-set Enrichment (GAGE) (v2.48.0) R package with the MSigDB gene-set repository as reference. transcription factor and pathway activity analyses using decoupleR (v2.5.3) 7 with Dorothea 8and Progeny 9 knowledge bases respectively.<br>Mapping of long read bulk sequencing data to scRNA was performed using Combat_seq model and DESeq2.

For manuscripts utilizing custom algorithms or software that are central to the research but not yet described in published literature, software must be made available to editors and reviewers. We strongly encourage code deposition in a community repository (e.g. GitHub). See the Nature Portfolio guidelines for submitting code & software for further information.

Data

Policy information about availability of data

All manuscripts must include a data availability statement. This statement should provide the following information, where applicable:

- Accession codes, unique identifiers, or web links for publicly available datasets
- A description of any restrictions on data availability
- For clinical datasets or third party data, please ensure that the statement adheres to our policy

Microglia and BTSC RNA-seq data have been deposited at GEO and are publicly available as of the date of publication. GEO accession numbers: GSE227722 (microglia) and GSE247079 (BTSC268 and BTSC475). Gene expression array in BTSC233 (GSE97347), JX6 (GSE97349) and SNB19 cells (GSE138890) have been previously described (Fedele et al., 2017; Masilamani et al., 2016).

Research involving human participants, their data, or biological material

Policy information about studies with human participants or human data. See also policy information about sex, gender (identity/presentation), and sexual orientation and race, ethnicity and racism.

Reporting on sex and gender	Patient-derived samples were chosen to contain a similar number of male and females. The role of the patient sex in the results could not be ruled out given the limited number of samples.
Reporting on race, ethnicity, or other socially relevant groupings	Information on race, ethnicity or other socially relevant groupings was not collected.
Population characteristics	Samples were obtained from patients with diagnosis of Glioma, grades I, II, III and IV. Patients male and females in the range between 2 and 74.
Recruitment	Department of Neurosurgery of the University Medical Center Freiburg (Freiburg, Germany)
Ethics oversight	University Medical Center Freiburg (Freiburg, Germany)

Note that full information on the approval of the study protocol must also be provided in the manuscript.

Field-specific reporting

Please select the one below that is the best fit for your research. If you are not sure, read the appropriate sections before making your selection.

☒ Life sciences ☐ Behavioural & social sciences ☐ Ecological, evolutionary & environmental sciences

For a reference copy of the document with all sections, see nature.com/documents/nr-reporting-summary-flat.pdf

Life sciences study design

All studies must disclose on these points even when the disclosure is negative.

Sample size	We considered a minumum of three biological replicates for performing a statistical analysis.
Data exclusions	There was no data exclusion.
Replication	Only experiments that could be reliably repeated were included in the study.
Randomization	Samples were allocated into different groups randomly.
Blinding	When possible, the investigator was blinded to group allocation when performing the analysis.

Behavioural & social sciences study design

All studies must disclose on these points even when the disclosure is negative.

Study description	*Briefly describe the study type including whether data are quantitative, qualitative, or mixed-methods (e.g. qualitative cross-sectional, quantitative experimental, mixed-methods case study).*
Research sample	*State the research sample (e.g. Harvard university undergraduates, villagers in rural India) and provide relevant demographic information (e.g. age, sex) and indicate whether the sample is representative. Provide a rationale for the study sample chosen. For studies involving existing datasets, please describe the dataset and source.*

Sampling strategy	*Describe the sampling procedure (e.g. random, snowball, stratified, convenience). Describe the statistical methods that were used to predetermine sample size OR if no sample-size calculation was performed, describe how sample sizes were chosen and provide a rationale for why these sample sizes are sufficient. For qualitative data, please indicate whether data saturation was considered, and what criteria were used to decide that no further sampling was needed.*
Data collection	*Provide details about the data collection procedure, including the instruments or devices used to record the data (e.g. pen and paper, computer, eye tracker, video or audio equipment) whether anyone was present besides the participant(s) and the researcher, and whether the researcher was blind to experimental condition and/or the study hypothesis during data collection.*
Timing	*Indicate the start and stop dates of data collection. If there is a gap between collection periods, state the dates for each sample cohort.*
Data exclusions	*If no data were excluded from the analyses, state so OR if data were excluded, provide the exact number of exclusions and the rationale behind them, indicating whether exclusion criteria were pre-established.*
Non-participation	*State how many participants dropped out/declined participation and the reason(s) given OR provide response rate OR state that no participants dropped out/declined participation.*
Randomization	*If participants were not allocated into experimental groups, state so OR describe how participants were allocated to groups, and if allocation was not random, describe how covariates were controlled.*

Ecological, evolutionary & environmental sciences study design

All studies must disclose on these points even when the disclosure is negative.

Study description	*Briefly describe the study. For quantitative data include treatment factors and interactions, design structure (e.g. factorial, nested, hierarchical), nature and number of experimental units and replicates.*
Research sample	*Describe the research sample (e.g. a group of tagged Passer domesticus, all Stenocereus thurberi within Organ Pipe Cactus National Monument), and provide a rationale for the sample choice. When relevant, describe the organism taxa, source, sex, age range and any manipulations. State what population the sample is meant to represent when applicable. For studies involving existing datasets, describe the data and its source.*
Sampling strategy	*Note the sampling procedure. Describe the statistical methods that were used to predetermine sample size OR if no sample-size calculation was performed, describe how sample sizes were chosen and provide a rationale for why these sample sizes are sufficient.*
Data collection	*Describe the data collection procedure, including who recorded the data and how.*
Timing and spatial scale	*Indicate the start and stop dates of data collection, noting the frequency and periodicity of sampling and providing a rationale for these choices. If there is a gap between collection periods, state the dates for each sample cohort. Specify the spatial scale from which the data are taken*
Data exclusions	*If no data were excluded from the analyses, state so OR if data were excluded, describe the exclusions and the rationale behind them, indicating whether exclusion criteria were pre-established.*
Reproducibility	*Describe the measures taken to verify the reproducibility of experimental findings. For each experiment, note whether any attempts to repeat the experiment failed OR state that all attempts to repeat the experiment were successful.*
Randomization	*Describe how samples/organisms/participants were allocated into groups. If allocation was not random, describe how covariates were controlled. If this is not relevant to your study, explain why.*
Blinding	*Describe the extent of blinding used during data acquisition and analysis. If blinding was not possible, describe why OR explain why blinding was not relevant to your study.*

Did the study involve field work? ☐ Yes ☐ No

Field work, collection and transport

Field conditions	*Describe the study conditions for field work, providing relevant parameters (e.g. temperature, rainfall).*
Location	*State the location of the sampling or experiment, providing relevant parameters (e.g. latitude and longitude, elevation, water depth).*
Access & import/export	*Describe the efforts you have made to access habitats and to collect and import/export your samples in a responsible manner and in compliance with local, national and international laws, noting any permits that were obtained (give the name of the issuing authority, the date of issue, and any identifying information).*
Disturbance	*Describe any disturbance caused by the study and how it was minimized.*

Reporting for specific materials, systems and methods

We require information from authors about some types of materials, experimental systems and methods used in many studies. Here, indicate whether each material, system or method listed is relevant to your study. If you are not sure if a list item applies to your research, read the appropriate section before selecting a response.

Materials & experimental systems

n/a	Involved in the study
☐	☒ Antibodies
☐	☒ Eukaryotic cell lines
☒	☐ Palaeontology and archaeology
☐	☒ Animals and other organisms
☒	☐ Clinical data
☒	☐ Dual use research of concern
☒	☐ Plants

Methods

n/a	Involved in the study
☒	☐ ChIP-seq
☒	☐ Flow cytometry
☒	☐ MRI-based neuroimaging

Antibodies

Antibodies used	The following antibodies were used:<br>anti-ZBTB18 antibody (Abcam #ab118471), lot GR3369487-2;<br>anti-Lamin A/C antibody (Cell Signaling #4777), clone 4C11,5;<br>anti-alpha Tubulin (Abcam, #ab7291), clone DM1A;<br>anti-IBA1 rabbit monoclonal (Abcam, #ab178846;), clone EPR16588. Lot GR3235737-4;<br>anti-ZBTB18 rabbit polyclonal Proteintech, (#12714-1-AP;), lot 00018520;<br>anti-F4/80 rat monoclonal (Invitrogen, MA1-91124,), clone A3-1, lot XJ3737322;<br>anti-MHC Class II rabbit polyclonal (Invitrogen, PA5-116876), lot YC3854371B;<br>anti-HA (Abcam, #18181), clone HA.C5;<br>anti-FLAG mouse monoclonal (Sigma #F1804, clone M2), lot 085M4774V;<br>anti-H3K4me2 rabbit polyclonal (Cell Signaling #9725), lot 13;<br>anti-GDF15 (Sigma, #AMAb90687), lot 02600;<br>anti-CCL2 (Bio-Techne), clone 23007;, lot Ju1423081<br>anti GDF15 (Bio-Techne), clone 147627, lotUDC1122111;<br>anti-LSD1 (santa Cruz), SC-54875, lot I1415.
Validation	All antibodies were tested by the manufacturers for the applications mentioned in our study.

Eukaryotic cell lines

Policy information about cell lines and Sex and Gender in Research

Cell line source(s)	Patient-derived glioblastoma stem cells-like (BTSC233, BTSC268 and BTSC475) were generated in our laboratory in accordance with an Institutional Review Board-approved protocol.The patient-derived JX6 GBM xenoline was previously described (Fedele et al., 2017; Masilamani et al., 2016). Patient-derived glioblastoma stem cells-like GBM#22 sgLSD1#1 were previously described (Faletti et al., 2021; Ferrarese et al., 2023).<br>Human immortalized microglia (ABM-T0251; Applied Biological Materials).<br>SNB19 and HEK293T were obtained from ATCC.
Authentication	SNB19 have been authenticated on 3/2/2017 and on 11/26/19 (SNB19 and HEK293T) by PCR-single-locus-technology (Eurofins Medigenomix).
Mycoplasma contamination	All the cells were mycoplasma free.
Commonly misidentified lines<br>(See ICLAC register)	SNB-19 (ATCC CRL-2219) was reported to have a STR pattern identical to that for GBM cells U-373 MG (ATCC HTB-17). Since we did not compare SNB19/U-373 to other established glioblastoma cell lines, this does not affect our results. Moreover, SNB19/U-373 cells were used only for limited experiments since we validated and expanded our findings in primary GBM-derived cells.

Animals and other research organisms

Policy information about studies involving animals; ARRIVE guidelines recommended for reporting animal research, and Sex and Gender in Research

Laboratory animals	Female 6 weeks old NOS/SCID mice (from Fedele et al., 2017); female 6 weeks old C57BL/6 mice

Wild animals	N/A
Reporting on sex	N/A
Field-collected samples	N/A
Ethics oversight	The study was approved by the local authorities.

Note that full information on the approval of the study protocol must also be provided in the manuscript.

Plants

Seed stocks	*Report on the source of all seed stocks or other plant material used. If applicable, state the seed stock centre and catalogue number. If plant specimens were collected from the field, describe the collection location, date and sampling procedures.*
Novel plant genotypes	*Describe the methods by which all novel plant genotypes were produced. This includes those generated by transgenic approaches, gene editing, chemical/radiation-based mutagenesis and hybridization. For transgenic lines, describe the transformation method, the number of independent lines analyzed and the generation upon which experiments were performed. For gene-edited lines, describe the editor used, the endogenous sequence targeted for editing, the targeting guide RNA sequence (if applicable) and how the editor was applied.*
Authentication	*Describe any authentication procedures for each seed stock used or novel genotype generated. Describe any experiments used to assess the effect of a mutation and, where applicable, how potential secondary effects (e.g. second site T-DNA insertions, mosiacism, off-target gene editing) were examined.*
